# Persistently Elevated Expression of Systemic, Soluble Co-Inhibitory Immune Checkpoint Molecules in People Living with HIV before and One Year after Antiretroviral Therapy

**DOI:** 10.3390/pathogens13070540

**Published:** 2024-06-27

**Authors:** Robyn-Brooke Labuschagne Naidoo, Helen C. Steel, Annette J. Theron, Ronald Anderson, Gregory R. Tintinger, Theresa M. Rossouw

**Affiliations:** 1 Department of Internal Medicine, School of Medicine, Faculty of Health Sciences, University of Pretoria and Steve Biko Academic Hospital, Pretoria 0002, South Africa; robynbrooke7@gmail.com (R.-B.L.N.); gregory.tintinger@up.ac.za (G.R.T.); 2 Department of Immunology, School of Medicine, Faculty of Health Sciences, University of Pretoria, Pretoria 0002, South Africa; helen.steel@up.ac.za (H.C.S.); atheron@up.ac.za (A.J.T.); ronald.anderson@up.ac.za (R.A.)

**Keywords:** antiretroviral treatment, HIV, co-inhibitory immune checkpoints, CTLA-4, LAG-3, PD-1, PD-L1, TIM-3

## Abstract

Introduction: Increasing drug resistance and the absence of a cure necessitates exploration of novel treatment strategies for people living with HIV (PLWH). Targeting of soluble co-inhibitory immune checkpoint molecules (sICMs) represents a novel, potentially effective strategy in the management of HIV. Methods: In this retrospective, longitudinal, observational study, the plasma levels of five prominent co-inhibitory sICMs—CTLA-4, LAG-3, PD-1 and its ligand PD-L1, as well as TIM-3—were quantified in 68 PLWH—before and one year after antiretroviral therapy (ART)—and compared with those of 15 healthy control participants. Results: Relative to control participants, PLWH had substantially elevated pre-treatment levels of all five co-inhibitory sICMs (*p* < 0.0001–*p* < 0.0657), which, over the 12-month period of ART, remained significantly higher than those of controls (*p* < 0.0367–*p* < 0.0001). PLWH with advanced disease, reflected by a CD4+ T cell count <200 cells/mm^3^ before ART, had the lowest levels of CTLA-4 and LAG-3, while participants with pre-treatment HIV viral loads ≥100,000 copies/mL had higher pre-treatment levels of TIM-3, which also persisted at 12 months. Conclusions: Plasma levels of CTLA-4, LAG-3, PD-1, PD-L1 and TIM-3 were significantly elevated in treatment-naïve PLWH and remained so following one year of virally-suppressive ART, possibly identifying LAG-3 and TIM-3 in particular as potential targets for adjuvant immunotherapy.

## 1. Introduction

HIV infects and kills CD4+ T cells and leads to progressive loss of these cells from the circulation, as well as depletion of total body stores [1]. The number of circulating CD4+ T cells in PLWH predicts the onset of overt immunodeficiency and acquired immunodeficiency syndrome (AIDS). Suppressing HIV replication rapidly increases peripheral blood CD4+ T cell counts and reverses the immunodeficiency, although this is often incomplete [2]. One of the reasons for persistent functional immunodeficiency is chronic systemic immune activation and resultant T cell exhaustion that persist despite successful antiretroviral therapy (ART) [3,4].

T cell activation is tightly regulated by co-signalling molecules known as co-stimulatory and co-inhibitory immune checkpoint molecules (ICMs). If major histocompatibility complex and T cell receptor binding is accompanied by the engagement of co-stimulatory molecules, this event allows T cells to proliferate and to migrate toward specific target cells, expanding the adaptive immune response. On the other hand, if binding is accompanied by the engagement of co-inhibitory receptors, T cell activation is suppressed to maintain self-tolerance and modulate the duration and amplitude of physiological immune responses in peripheral tissues to minimise collateral tissue damage [5].

During chronic antigenic stimulation, such as during chronic viral infections, T cells undergo distinct epigenetic and metabolic changes that confer immune exhaustion. In this state, T cells have increased expression of co-inhibitory ICMs and a reduced capacity to secrete cytokines [6]. There is a sequential loss of T cell effector functions in a hierarchical manner, with loss of interleukin (IL)-2 production being the earliest sign. Factors such as the duration and magnitude of antigenic activation, levels of stimulatory or suppressive cytokines and the expression and diversity of co-stimulatory or co-inhibitory ICMs determine the severity of the exhaustion [3].

Exhausted T cells can express a variety of inhibitory checkpoints. Cytotoxic T-lymphocyte associated antigen 4 (CTLA-4), lymphocyte-activation gene 3 protein (LAG-3), programmed cell death protein 1 (PD-1), programmed death protein ligand 1 (PD-L1) and cytotoxic T cell immunoglobulin and mucin-domain 3 (TIM-3) have all been shown to play a role in chronic viral persistence and are often used as markers to define exhausted T cells during HIV [7,8].

Cancer cells also upregulate expression of co-inhibitory ICMs. In this context, tumour cells expressing PD-L1 induce immune cell tolerance, thereby facilitating tumour progression. This has led to treatment strategies based on the implementation of immunotherapies, which stimulate the immune system to participate in immunosurveillance, attenuate immune evasion and destroy cancer cells by means of immune checkpoint blockade [9]. Fully-human and humanised monoclonal antibodies have been developed to impede the interaction of the prominent co-inhibitory ICMs, CTLA-4 and PD-1 with their ligands, resulting in improved T cell proliferation and upregulated host immune responses against cancer cells [10].

Whether similar therapeutic responses can be achieved with chronic viral infections is currently unclear, but the success achieved with this approach in the oncology field suggests that possible future treatment plans using the same immune principles in chronic viral infections and the resultant exhausted state need to be carefully evaluated. To date, however, limited work has been done to assess the clinical consequences of ICM blockade in HIV infection.

Importantly, soluble, cell-free ICMs are generated intracellularly by alternative mRNA splicing or, alternatively, from the surface of cells—most prominently those of the adaptive and innate immune systems—by proteolytic cleavage [11]. These systemic, soluble variants of co-inhibitory ICMs not only retain immunosuppressive properties but may also attenuate the therapeutic potential of checkpoint-targeted monoclonal antibodies [11,12]. Importantly, prior detection of these systemic, co-inhibitory sICMs may also guide immunotherapy [13].

To the best of our knowledge, no studies have characterised the expression of systemic, soluble, co-inhibitory ICMs in African PLWH, a population with higher background antigenic exposure and potentially higher levels of T cell exhaustion. For this reason, we set out to study the expression of soluble, systemic, co-inhibitory ICMs in PLWH, before and after successful ART, in South Africa, the country with the largest population of PLWH in the world.

## 2. Methods

Sixty-eight blood samples from treatment-naïve PLWH who initiated a fixed dose ART combination that consisted of tenofovir disoproxil fumarate (TDF), lamivudine (3TC) and efavirenz (EFV) were randomly selected from a longitudinal, observational study that was conducted between March 2014 and August 2015. Clinical data were collected from participants at Eersterust Community Health Centre in Tshwane, South Africa. All participants had achieved a satisfactory virological treatment response (HIV viral load ≤1000 copies/mL). Fifteen samples collected from healthy volunteers in the Department of Immunology, University of Pretoria, formed the control group of participants not living with HIV. All participants had given written informed consent for participation and the study was approved by the Research Ethics Committee of the Faculty of Health Sciences at the University of Pretoria (ethics number: 358/2021).

Levels of the five prominent systemic, co-inhibitory soluble ICMs (sICMs) for which targeted immunotherapy is available were measured in plasma before ART initiation and after 12 months of treatment using a custom Milliplex^®^ MAP kit (Merck KGaA, Darmstadt, Germany). The kit included five ICMs: CTLA-4, LAG-3, PD-1, PD-L1 and TIM-3.

Briefly, the stored frozen (−80 °C) plasma samples were thawed at room temperature and then vortex-mixed. To each well of a 96-well microplate, 50 µL of the magnetic bead mixture was added. Thereafter the plate was washed twice using an automated magnetic plate washer (Bio-Rad Laboratories Inc., Hercules, CA, USA). A universal assay buffer (25 μL) was added to each well, followed by 25 µL of prepared standards, samples, controls and blanks to the appropriately assigned wells. The plate was then sealed and incubated for 2 h. All incubations were carried out at room temperature with gentle agitation on an orbital shaker (Stuart Scientific, Johannesburg, South Africa), and the plate was protected from light. Following incubation, the assay plate was washed twice (as described above). Detection antibodies (25 µL) were added to each well and the plate was incubated (as described above) for an additional 30 min. After this incubation period, the plate was washed (as described above) and 25 µL of streptavidin R-phycoerythrin conjugate were added to each well. The plate was incubated with agitation as described above for an additional 30 min followed by a final two washes (as described above). Reading buffer (120 µL) was added to each well and the plate sealed and shaken vigorously for 2 min using a microplate shaker (Thomas Scientific, Swedesboro, NJ, USA) to ensure that the beads were resuspended. The biomarkers were assayed using a Bio-Rad Luminex^®^ 200™ Suspension Array System (Bio-Rad Laboratories, Inc.). Bio-Manager Software 6.0 was used for bead acquisition and analysis of median fluorescence intensity. The results were recorded as picograms (pg)/mL.

Blood cotinine levels, as biomarkers of tobacco usage, were available in a subset of 32 participants. A level <15 nanograms (ng)/mL was taken as negative and a level ≥15 ng/mL was taken as a positive indication of active tobacco use [14].

Data were entered in a Microsoft Excel spreadsheet. A double-entry method was used to avoid data entry errors. In addition, data were visually inspected by means of histograms to detect any outliers. Data were exported to Stata 17 (StataCorp, Stata Corp (2023) Stata Statistical Software: Release 17. College Station, TX, USA: StataCorp LLC) for analysis. Data were described by means of frequencies, proportions, medians and interquartile ranges. Associations were tested by means of the Kruskal–Wallis test for continuous variables and Pearson’s chi-square or Fisher’s exact test for categorical variables, as appropriate. Correlations were tested with the Spearman correlation test with Bonferroni correction for multiple comparisons. Changes within the group over time were assessed with the Wilcoxon rank sum test. A *p*-value less than 0.05 was considered to be statistically significant, and *p*-values below 0.1 were noted as potential future avenues of exploration.

## 3. Results

The PLWH comprised 42 (61.8%) females and 26 (38.2%) males. The median age was 38.3 years (interquartile range (IQR) 31.9–44.9; range 21.5–69.6). Control participants had a similar sex distribution: 61.5% females and 38.5% males, with a median age of 42 years (IQR 35–50 years).

### 3.1. CD4+ Cell Counts and Viral Loads

The CD4 counts and HIV VL results of PLWH before and after 12 months of ART are shown in Table 1. Before treatment, 53.7% (36/37—one missing value) of PLWH had a CD4 count <200 cells/mm^3^ and this decreased to 17.7% (12/68) after 12 months of ART. The CD4:CD8 ratio was very low at both time points with a single patient having a ratio above 1 before ART and 11 (16.2%) patients after 12 months of ART. In keeping with this extent of immunodeficiency, the HIV VL was high before ART with 57.4% (39/68) of PLWH having a VL ≥ 100 000 copies/mL at this time point.

### 3.2. Comparison of Plasma Concentrations of the Test Soluble Co-Inhibitory Immune Checkpoints between PLWH and Control Participants, as well as before and after ART

As shown in Table 2, prior to administration of ART, PLWH had significantly higher levels of four of the sICMs, namely LAG-3, PD-1, PD-L1 and TIM-3 (*p* < 0.0030–*p* < 0.0001), while that of CTLA-4 was numerically higher without achieving statistical significance (*p* < 0.0657). Over the 12-month period of ART, the plasma concentrations of four sICMs remained unchanged from the baseline values: CTLA-4 (*p* = 0.6752), LAG-3 (*p* = 0.1077), PD-1 (*p* = 0.8938) and PD-L1 (*p* = 0.2073), while that of TIM-3 decreased moderately, albeit significantly, following ART (*p* = 0.0001). All sICMs remained significantly higher in comparison with the corresponding values for the group of healthy control participants (*p* = 0.0011).

There were no differences in plasma levels of the soluble ICMs between male and female participants either before (Supplementary Table S1) or after 12 months of ART (Supplementary Table S2). Over the 12 months, men had a non-significantly greater decrease in TIM-3 levels than women (*p* = 0.0794).

### 3.3. Impact of Disease Severity on the Plasma Concentrations of the Soluble Co-Inhibitory Immune Checkpoints

Disease severity was measured by CD4+ T cell count and HIV viral load. Levels of CTLA-4 and LAG-3 were significantly lower in participants with a pre-treatment CD4+ T cell count of <200 cells/mm^3^ relative to participants with CD4+ counts ≥200 cells/mm^3^. After 12 months of ART, the only significant difference was LAG-3, with higher levels observed in participants with a pre-treatment CD4+ T cell count <200 cells/mm^3^. Over the 12-month period, levels of LAG-3 decreased in the higher CD4+ category group, but increased in the group with lower CD4+ T cell counts, and this difference was statistically significant (*p* = 0.0008). Pre-treatment CD4+ T cell count, as well as the CD4:CD8 ratio, were positively correlated with pre-treatment LAG-3 levels (rho = 0.375; *p* = 0.0018 and rho = 0.251; *p* = 0.0404) while CD4+ T cell counts were negatively correlated with the change in LAG-3 concentrations over 12 months (rho = −0.292; *p* = 0.0165).

A weak correlation persisted between the pre-treatment LAG-3 level and the 12-month CD4+ T cell count (rho = 0.245; *p* = 0.0440). No significant correlations were found between any of the sICMs after one year of treatment and the 12-month CD4+ T cell count.

Before ART, TIM-3 levels were significantly higher in participants with a pre-treatment viral load ≥100 000 copies/mL (*p* = 0.0356). After 12 months of treatment, TIM-3 concentrations were significantly higher in this group (*p* = 0.0397). Over the 12 months of treatment, PD-1 and PD-L1 levels increased in participants with a viral load <100,000 copies/mL, but decreased in those in the higher viral load category (*p* = 0.0465; *p* = 0.0261). No significant correlations were found between any of the ICMs and viral load.

### 3.4. Effects of Tobacco Usage on the Plasma Concentrations of the Soluble Co-Inhibitory Immune Checkpoints in PLWH before and after ART

Cotinine levels were available for 32 participants. Of these, 12 participants were using tobacco products and 20 were not. Nine of the tobacco users were women and three were men; however, there was no difference in the proportions of women (9/24–37.5%) and men (3/8–37.5%) who used tobacco (*p* = 0.999). Pre-treatment CD4 counts and viral loads were similar between the groups (*p* = 0.2816 and *p* = 0.2666, respectively). No differences were observed in the plasma concentrations of any of the five sICMs between the groups before ART; however, after 12 months, TIM-3 was significantly higher in tobacco users compared to non-users (Table 3).

Over the 12 months of ART, levels of CTLA-4, PD-1 and PD-L1 increased in tobacco users, while decreasing in non-users, with these differences attaining statistical significance (*p* = 0.0434, *p* = 0.0108 and *p* = 0.0309, respectively). Non-users had a significant decrease in expression of TIM-3 (*p* = 0.0010) while tobacco users had a very small decrease, but this difference in the change over 12 months did not reach statistical significance (*p* = 0.0928) (Supplementary Table S3).

### 3.5. Correlations between the Plasma Concentrations of the Various Soluble Co-Inhibitory Immune Checkpoints before and after ART

Before treatment, there were strong and significant correlations between CTLA-4 and PD-1 (rho = 0.907, *p* < 0.0001), CTLA-4 and PD-L1 (rho = 0.895, *p* < 0.0001) and PD-L1 and PD-1 (rho = 0.873, *p* < 0.0001). There were significant but weak correlations between CTLA-4 and LAG-3 (rho = 0.450, *p* = 0.0001), as well as between LAG-3 and PD-1 (rho = 0.463, *p* = 0.0001) and LAG-3 and PD-L1 (rho = 0.437, *p* = 0.0002). TIM-3 was not significantly correlated with any of the other sICMs (Table 4). The correlations were unchanged after 12 months of ART. Age was not correlated with any of the ICMs at either time point (Supplementary Table S4).

## 4. Discussion

The current study has revealed that relative to healthy control participants, PLWH have substantially elevated plasma levels of the soluble, cell-free variants of five major co-inhibitory immune checkpoint molecules, notably CTLA-4, LAG-3, PD-1, PD-L1 and TIM-3. Somewhat concerningly and unexpectedly, however, administration of virally-suppressive ART for a one-year period, while effectively and significantly increasing and decreasing circulating CD4+ T cell counts and HIV viral loads, respectively, was not accompanied by detectable decreases in the plasma levels of any of the five test systemic, co-inhibitory sICMs. This observation appears to be consistent with a very slow recovery of immune competence following initiation of ART. Although reports are few, it is noteworthy that Avendaño-Ortiz et al., in agreement with the current study, have also described the presence of persistently high levels of sPD-L1 in plasma as being a prominent biomarker of both HIV-1 infection and virological failure [15].

Our findings are, however, in contrast to reports by Zilber et al. [16] and, more recently, by Chiu et al. [17]. In a study of male participants initiated a median of 46 days after estimated seroconversion, Zilber et al. [16] demonstrated that levels of sPD-1 and sTIM-3 were significantly higher in PLWH when compared to healthy controls, but had decreased to levels similar to controls after one year of ART. Chiu et al. [17] reported that the median levels of sLAG-3, sPD-1 and sPD-L2 were higher in PLWH compared to HIV-uninfected controls, but that levels of sLAG-3 and sPD-1 were significantly lower in those on virally-suppressive ART and similar to HIV-uninfected controls. Apart from the obvious geographic differences between the above-mentioned studies and the current study, our participants started with much lower CD4 counts (median 184 (IQR 74–317) versus 526 (IQR 405.2–667.8) [16] versus 362 (IQR 212.5–527.5) [17] cells/mm^3^). Another important difference is the timing and duration of ART. While time of seroconversion was not available for our participants, the low CD4 counts suggest late introduction of ART. In fact, 54% of participants in our study had a CD4 count <200 cells/mm^3^. In addition, participants had been on ART for only one year compared to three years in the study reported by Chiu et al. [17]. In this context, it is well known that late initiation of ART is accompanied by greater levels of immune activation and exhaustion, as well as incomplete recovery of CD4+ and CD8+ T cell function [18]. It is still unknown, however, whether persistent elevation of sICMs might be contributing to this exhausted phenotype.

In the current study, exploration of possible relationships of the pre-treatment CD4 cell counts and viral loads with the plasma levels of five sICMs did, however, reveal significant associations. First, categorisation of PLWH according to CD4 counts revealed that those with more advanced disease, associated with CD4 counts of <200 cells/mm^3^, had significantly lower levels of CTLA-4 and LAG-3. Notably, both of these checkpoints are highly expressed on regulatory T cells (Tregs) [19], which are selectively decreased during progression of HIV infection, possibly as a strategy to counter the rate of immune dysfunction, or, more likely, due to the predilection of these cells as a target of HIV [20]. Secondly, those PLWH with pre-treatment viral loads of ≥100,000 copies/mL exhibited higher plasma levels of TIM-3, which persisted throughout the one-year period of administration of ART. The broad expression of TIM-3 on various types of immune cells, including CD4+ and CD8+ T cells, may account for the increased levels of this checkpoint molecule [21]. Additionally, the significant, selective association of this co-inhibitory sICM may identify it as a potential target for immunotherapy in HIV infection.

The use of immune checkpoint blockade has gained traction in the HIV cure field over the past few years. The interest is two-fold: firstly, checkpoint blockade could help reverse latency through activating HIV expression in latently-infected cells and, secondly, it may enhance HIV-specific T cell function by revitalising exhausted T cells [8]. Progress has been hampered, however, by the toxicity of the currently available mAbs targeting PD-1, PD-L1 and CTLA-4. Especially concerning is the high frequency of immune-related adverse events, such as thyroiditis, hepatitis [22] and auto-immune hypophysitis [23], indicating that PLWH might be especially prone to toxicities due to HIV-induced immune dysregulation [24].

Innovative strategies that incorporate different approaches, such as low-dose administration of a combination of immune checkpoint-targeted antibodies, have been proposed [8]. In the case of HIV infection, combination therapy has been the cornerstone of HIV treatment, not only in terms of ART but also with broadly-neutralizing mAb therapy [25]. In the context of immune checkpoint blockade, ICMs that are also expressed on innate immune cells could play an important synergistic role in such a combination strategy. TIM-3, for instance, is not only expressed on IFN-γ-producing T cells and FoxP3+ Treg cells, but also on macrophages and dendritic cells, suppressing their responses upon interaction with their ligands [26]. In our study, TIM-3 was not correlated with any of the other four ICMs, possibly indicating an independent expression profile. As expected, PD-1 was strongly correlated with its ligand, PD-L1, while LAG-3 was weakly correlated with CTLA-4, PD-1 and PD-L1, suggesting that LAG-3 and CTLA-4 might also be useful in combination strategies with PD-1. The potential utility of TIM-3 and LAG-4 seems to be relatively unexplored in comparison to that of PD-1/PD-L1 and CTLA-4. As opposed to mAb targeting of co-inhibitory immune checkpoints, future innovations include the utility of agonists of co-stimulatory immune checkpoints such as vopratelimab, a humanised mAb which activates inducible T cell co-stimulator (ICOS) expressed on primed T cells [27] and novel small molecule inhibitors of PD-1/PD-L1 [28]. In this context, evaluation of the levels of soluble co-stimulatory checkpoints would be a valuable pre-requisite and a potentially important extension of the current study.

Finally, PLWH who use tobacco are known to have increased AIDS-related and non-AIDS-related mortality [29], worse HIV treatment control [30] and higher levels of T cell exhaustion than non-tobacco users [31]. The findings of elevated levels of TIM-3 as well as increases in CTLA-4 and PD-L1 over the 12-month period are concerning, and particular attention should be paid to this population, especially with regards to tobacco use cessation strategies.

## 5. Conclusions

The current study was focused on identifying novel, potentially targetable chronic mechanisms of immunosuppression which characterise HIV infection. Our findings have demonstrated a concerning persistence of seemingly high levels of immune dysfunction in PLWH with advanced disease, even in the face of virally-suppressive ART. Notwithstanding the potential of immune checkpoint blockade in HIV cure strategies, this study further highlights the urgent need for novel immunotherapeutic strategies as adjuncts to ART, as well as the necessity of conducting these kinds of studies in geographically diverse settings, including in Africa where late presentation to care is commonplace.

## Figures and Tables

**Table 1:** Comparison of CD4 counts and HIV viral loads of PLWH before and after ART.

Variable	PLWH before ART N = 68	PLWH at 12 Months N = 68	*p*-Value
CD4 count (cells/mm^3^)	184 (74–317) *	432 (245–582)	**<0.0001**
CD4 percentage (%)	11.07 (6.47–18.59) *	20.22 (13.24–31.01)	**<0.0001**
CD4:CD8 ratio	0.23 (0.09–0.42) *	0.48 (0.28–0.85)	**<0.0001**
HIV VL (copies/mL)	166,000 (27,000–560,000)	50	**<0.0001**
HIV VL (log)	5.2 (4.2–5.7)	1.7	**<0.0001**

* One participant did not have a pre-treatment CD4 count available; all values are shown as medians (interquartile range); bold indicates significance. N = number.

**Table 2:** Comparison of immune checkpoint levels in PLWH before and after ART and with people without HIV (Controls).

Variable	PLWH before ART N = 68	PLWH at 12 Months N = 68	Control N = 15	*p*-Value	
				Pre-ART vs. Controls	On ART vs. Controls
CTLA-4	316.84 (106.92–622.97)	342.50 (93.22–639.99)	0.74 (0.73–1323.79)	0.0657	**0.0367**
LAG-3	213,191.10 (154,145.20–256,134.90)	232,113.60 (178,068.40–319,052.60)	4293.78 (162.81–45,602.51)	**0.0001**	**0.0001**
PD-1	3486.50 (2013.10–6240.06)	3571.70 (1670.56–6515.27)	849.36 (154.96–1869.33)	**0.0011**	**0.0033**
PD-L1	628.37 (270.09–1094.94)	771.59 (235.26–1316.53)	101.02 (33.26–294.59)	**0.0030**	**0.0017**
TIM-3	3450.73 (2449.28–4884.63)	2817.07 (2141.62–3762.79)	128.14 (4.90–2936.02)	**0.0001**	**0.0011**

Abbreviations: People living with HIV (PLWH), Cytotoxic T-lymphocyte-associated antigen 4 (CTLA-4), Lymphocyte-activation gene 3 (LAG-3), Number (N), Programmed cell death protein 1 (PD-1), Programmed death protein ligand 1 (PD-L1), T cell immunoglobulin and mucin-domain 3 (TIM-3); all values are in pg/mL and shown as median (IQR); bold indicates significance.

**Table 3:** Association between tobacco use status and co-inhibitory immune checkpoint levels in PLWH after 12 months of ART.

Variable	Non-Users N = 20	Tobacco Users N = 12	*p*-Value
CTLA-4	315.91 (93.22–692.07)	370.27 (152.30–779.56)	0.3485
LAG-3	226,348.30 (179,752.60–277,634.30)	203,324.00 (187,789.00–283,333.90)	0.4381
PD-1	3289.34 (1490.30–5864.21)	4082.90 (2221.17–7413.38)	0.1959
PD-L1	568.52 (267.93–1281.22)	776.14 (369.52–1259.48)	0.3064
TIM-3	2565.10 (1932.08–3079.84)	3330.80 (2693.80–3893.81)	**0.0169**

Abbreviations: People living with HIV (PLWH), Cytotoxic T-lymphocyte-associated antigen 4 (CTLA-4), Lymphocyte-activation gene 3 (LAG-3), Number (N), Programmed cell death protein 1 (PD-1), Programmed death protein ligand 1 (PD-L1), T cell immunoglobulin and mucin-domain 3 (TIM-3); all values are in pg/mL and shown as median (IQR); bold indicates significance.

**Table 4:** Correlations among immune checkpoint molecules before ART in PLWH.

	CTLA-4	LAG-3	PD-1	PD-L1	TIM-3
**CTLA-4**	1.0				
**LAG-3**	0.450 **(0.0001)**	1.0			
**PD-1**	0.907 **(<0.0001)**	0.463 **(0.0001)**	1.0		
**PD-L1**	0.895 **(<0.0001)**	0.437 **(0.0002)**	0.873 **(<0.0001)**	1.0	
**TIM-3**	0.081 (0.5100)	−0.053 (0.6668)	0.120 (0.3306)	0.088 (0.4769)	1.0

Data presented as Spearman’s rho with *p*-value in brackets; bold indicates significance.

## Data Availability

The data that support the findings of this study are available from the corresponding author upon reasonable request.

## Supplementary Materials

The following supporting information can be downloaded at: https://www.mdpi.com/article/10.3390/pathogens13070540/s1, Table S1: Comparison of soluble immune checkpoint levels between female and male PLWH before ART; Table S2: Comparison of soluble immune checkpoint levels between female and male PLWH after 12 months of ART; Table S3: Comparison of the change in soluble immune checkpoint levels before and after 12 months of ART between tobacco-users and non-users; Table S4: Correlations between age and soluble immune checkpoint molecules before treatment in PLWH.

## List of Abbreviations

3TC	lamivudine
AIDS	acquired immunodeficiency syndrome
ART	antiretroviral therapy
CTLA-4	cytotoxic T-lymphocyte-associated protein 4
EFV	efavirenz
HIV	Human immunodeficiency virus
ICM	immune checkpoint molecules
IFN-γ	interferon gamma
IL	interleukin
IQR	interquartile range
LAG-3	lymphocyte-activation gene 3 protein
mAb	monoclonal antibody
ng	nanogram
PD-1	programmed cell death protein 1
PD-L1	programmed cell death protein ligand 1
pg	picogram
PLWH	people living with HIV
sICM	soluble immune checkpoint molecules
TDF	tenofovir disoproxil fumarate
TIM-3	T cell immunoglobulin and mucin-domain 3
Tregs	regulatory T cells
