# Supplementary material for: Persistently Elevated Expression of Systemic, Soluble Co-Inhibitory Immune Checkpoint Molecules in People Living with HIV before and One Year after Antiretroviral Therapy
Source: Pathogens. 2024 Jun 27;13(7):540. doi: 10.3390/pathogens13070540 (PMC11279922; doi:10.3390/pathogens13070540)
Supplement: Supplementary file 1 [file pathogens-13-00540-s001.zip › pathogens-3045370-supplementary.pdf]

Supplementary Table S1. Comparison of soluble immune checkpoint levels between female and male PLWH before ART

Variable	Female PLWH before ART N = 42	Male PLWH before ART N = 26	p-value
CTLA-4	235.82 (105.82 - 635.30)	396.42 (108.76 - 621.55)	0.4642
LAG-3	208 083.60 (144 284.70 - 256 015.20)	220 275.40 (178 559.70 - 261 506.70)	0.6771
PD-1	3 425.13 (1 896.48 - 5 580.56)	4 292.12 (2 097.58 – 7 106.36)	0.4565
PD-L1	535.44 (255.37 - 1 092.75)	711.13 (312.15 - 1 458.59)	0.3602
TIM-3	3 570.25 (2 734.52 - 5 012.37)	3 166.79 (2 286.37 - 4 813.24)	0.5874

Abbreviations: People living with HIV (PLWH), Cytotoxic T-lymphocyte-associated antigen 4 (CTLA-4), Lymphocyte-activation gene 3 (LAG-3), Programmed cell death protein 1 (PD-1), Programmed death protein ligand 1 (PD-L1), T cell immunoglobulin and mucin-domain 3 (TIM-3).
All values are in pg/mL and shown as median (IQR).

Supplementary Table S2. Comparison of soluble immune checkpoint levels between female and male PLWH after 12 months of ART

Variable	Female PLWH after 12 months of ART N = 42	Male PLWH after 12 months of ART N = 26	p-value
CTLA-4	325.32 (120.11 - 590.98)	378.51 (65.45 - 779.45)	0.9095
LAG-3	232 113.60 (186 169.00 - 286 560.10)	227 493.70 (161 298.90 - 343 187.40)	0.8400
PD-1	3 484.34 (2 189.72 - 5 435.99)	4 345.67 (1 590.40 - 7 527.78)	0.7910
PD-L1	704.98 (299.16 - 1 213.30)	912.12 (182.99 - 1 389.49)	0.8896
TIM-3	3 085.16 (2 272.05 - 3 840.53)	2 586.95 (1 844.48 - 3 497.60)	0.1872

Abbreviations: People living with HIV (PLWH), Cytotoxic T-lymphocyte-associated antigen 4 (CTLA-4), Lymphocyte-activation gene 3 (LAG-3), Programmed cell death protein 1 (PD-1), Programmed death protein ligand 1 (PD-L1), T cell immunoglobulin and mucin-domain 3 (TIM-3).
All values are in pg/mL and shown as median (IQR).

Supplementary Table S3. Comparison of the change in soluble immune checkpoint levels before and after 12 months of ART between tobacco-users and non-users

Variable	Non-users N = 20	Tobacco users N = 12	p-value
CTLA-4	-152.65 (-333.88 – 78.99)	95.99 (55.47 – 236.54)	**0.0434**
LAG-3	8 113.01 (-33 694.51 – 101 335.40)	55 428.03 (-7 900.50 – 150 692.70)	0.2541

PD-1	-1 492.62 (-3 421.00 – 476.13)	1 123.47 (650.60 – 3 630.24)	**0.0108**
PD-L1	-75.84 (-420.46 – 159.82)	340.01 (43.79 – 754.23)	**0.0309**
TIM-3	-831.33 (-1 473.65 - -151.19)	-29.55 (-1 176.67 – 235.55)	0.0928

Abbreviations: People living with HIV (PLWH), Cytotoxic T-lymphocyte-associated antigen 4 (CTLA-4), Lymphocyte-activation gene 3 (LAG-3), Programmed cell death protein 1 (PD-1), Programmed death protein ligand 1 (PD-L1), T cell immunoglobulin and mucin-domain 3 (TIM-3).
All values are in pg/mL and shown as median (IQR).

Supplementary Table S4. Correlations between age and soluble immune checkpoint molecules before treatment in PLWH

	**CTLA-4**	**LAG-3**	**PD-1**	**PD-L1**	**TIM-3**
**Age**	0.058 (0.6393)	0.003 (0.9778)	0.044 (0.7221)	0.08 (0.4700)	0.061 (0.6209)

Abbreviations: People living with HIV (PLWH), Cytotoxic T-lymphocyte-associated antigen 4 (CTLA-4), Lymphocyte-activation gene 3 (LAG-3), Programmed cell death protein 1 (PD-1), Programmed death protein ligand 1 (PD-L1), T cell immunoglobulin and mucin-domain 3 (TIM-3).
Date presented as Spearman’s rho with p-value in brackets
